# Erythropoietin-PLGA-PEG as a local treatment to promote functional recovery and neurovascular regeneration after peripheral nerve injury

**DOI:** 10.1186/s12951-022-01666-5

**Published:** 2022-10-28

**Authors:** Kristen M. Manto, Prem Kumar Govindappa, Brandon Martinazzi, Aijie Han, John P. Hegarty, Zachary Koroneos, M. A. Hassan Talukder, John C. Elfar

**Affiliations:** 1 Department of Orthopaedics and Rehabilitation, The Pennsylvania State University College of Medicine, Hershey, PA 17033 USA; 2 Department of Materials Science and Engineering, The Pennsylvania State University, University Park, PA 16802 USA; 3 Department of Orthopaedics and Sports Medicine, University of Arizona College of Medicine, Tucson, AZ 85724 USA

**Keywords:** Peripheral nerve, Sciatic nerve, Crush injury, Thermogel, Block copolymer, Erythropoietin, PLGA, PEG, Angiogenesis

## Abstract

**Background:**

Traumatic peripheral nerve injury (TPNI) is a major medical problem with no universally accepted pharmacologic treatment. We hypothesized that encapsulation of pro-angiogenic erythropoietin (EPO) in amphiphilic PLGA-PEG block copolymers could serve as a local controlled-release drug delivery system to enhance neurovascular regeneration after nerve injury.

**Methods:**

In this study, we synthesized an EPO-PLGA-PEG block copolymer formulation. We characterized its physiochemical and release properties and examined its effects on functional recovery, neural regeneration, and blood vessel formation after sciatic nerve crush injury in mice.

**Results:**

EPO-PLGA-PEG underwent solution-to-gel transition within the physiologically relevant temperature window and released stable EPO for up to 18 days. EPO-PLGA-PEG significantly enhanced sciatic function index (SFI), grip strength, and withdrawal reflex post-sciatic nerve crush injury. Furthermore, EPO-PLGA-PEG significantly increased blood vessel density, number of junctions, and myelinated nerve fibers after injury.

**Conclusion:**

This study provides promising preclinical evidence for using EPO-PLGA-PEG as a local controlled-release treatment to enhance functional outcomes and neurovascular regeneration in TPNI.

**Supplementary Information:**

The online version contains supplementary material available at 10.1186/s12951-022-01666-5.

## Background

Peripheral nerve injury (PNI) is a significant medical issue occurring in approximately 3% of all trauma patients [1–3]. PNI can lead to severe and long-term physiological and functional consequences with diminished quality of life, with outcomes ranging from mild discomfort to limb paralysis and amputation. There are multiple and distinct causes of PNI, mainly mechanical or traumatic, vascular or ischemic, and chemical or neurotoxic [4]. The common clinically encountered crush injury is both a traumatic and vascular injury, causing a range of neuronal damage [5, 6]. Mild injuries can recover spontaneously, while others require prompt surgical intervention [7]. Regeneration of peripheral nerves does not always lead to full functional recovery and surgical methods are still the most reliable options for treatment as there are no current standard pharmacologic treatments [8]. But despite microsurgical advances, in many cases, significant deficits remain. Thus, there is an unmet clinical need for a therapeutic agent which can enhance functional recovery after PNI.

The vascular system of peripheral nerves is fragile and injury can lead to vascular destructions, hemorrhage, and hypoxia within the nerve sheaths [9, 10]. In response to these microvascular lesions, inflammatory reactions increase vascular permeability and subsequent intraneural oedema [11]. After an injury to the neural vascular system, neovascularization is vital to survival, growth, and regeneration of axons through the transport of several growth factors, cytokines, and various cell types such as Schwann cells, the cells crucial to peripheral nerve regeneration and remyelination [12]. Vascularization decreases fibroblast infiltration and resulting scar formation and provides an optimal nutritional environment for nerve regeneration [13]. In addition, there is growing compelling evidence for the interplay between blood vessels and peripheral nerves and the alignment of regenerating nerves with blood vessels.

In an effort to promote angiogenesis after nerve injury, we used erythropoietin (EPO), a pleiotropic hormone approved by the U.S. Food and Drug Administration (FDA) for anemia treatment. EPO is an endogenous stimulant of vessel growth, with studies supporting its effects on endothelial cell proliferation and vasculogenesis [14, 15]. In addition to its effects as a pro-angiogenic factor, EPO has potent anti-inflammatory, anti-apoptotic, anti-oxidative, phagocytic, neurotrophic, and neuroprotective effects [15, 16]. In repurposing studies, EPO has been shown to enhance functional recovery after PNI in mice by improving sciatic function index (SFI) [17, 18]. It has also been shown to contribute to early angiogenic responses following nerve injury [18]. Despite its efficacy, the use of EPO may garner safety concerns due to systemic side effects such as flu-like symptoms, increased blood viscosity, hypertension, and thrombosis [19]. Because EPO has direct effects on blood parameters, patients also require hematologic monitoring. We hypothesized that a local formulation of EPO could help improve patient compliance, reduce adverse effects, and minimize the need for hematologic monitoring.

**Fig. 1:**
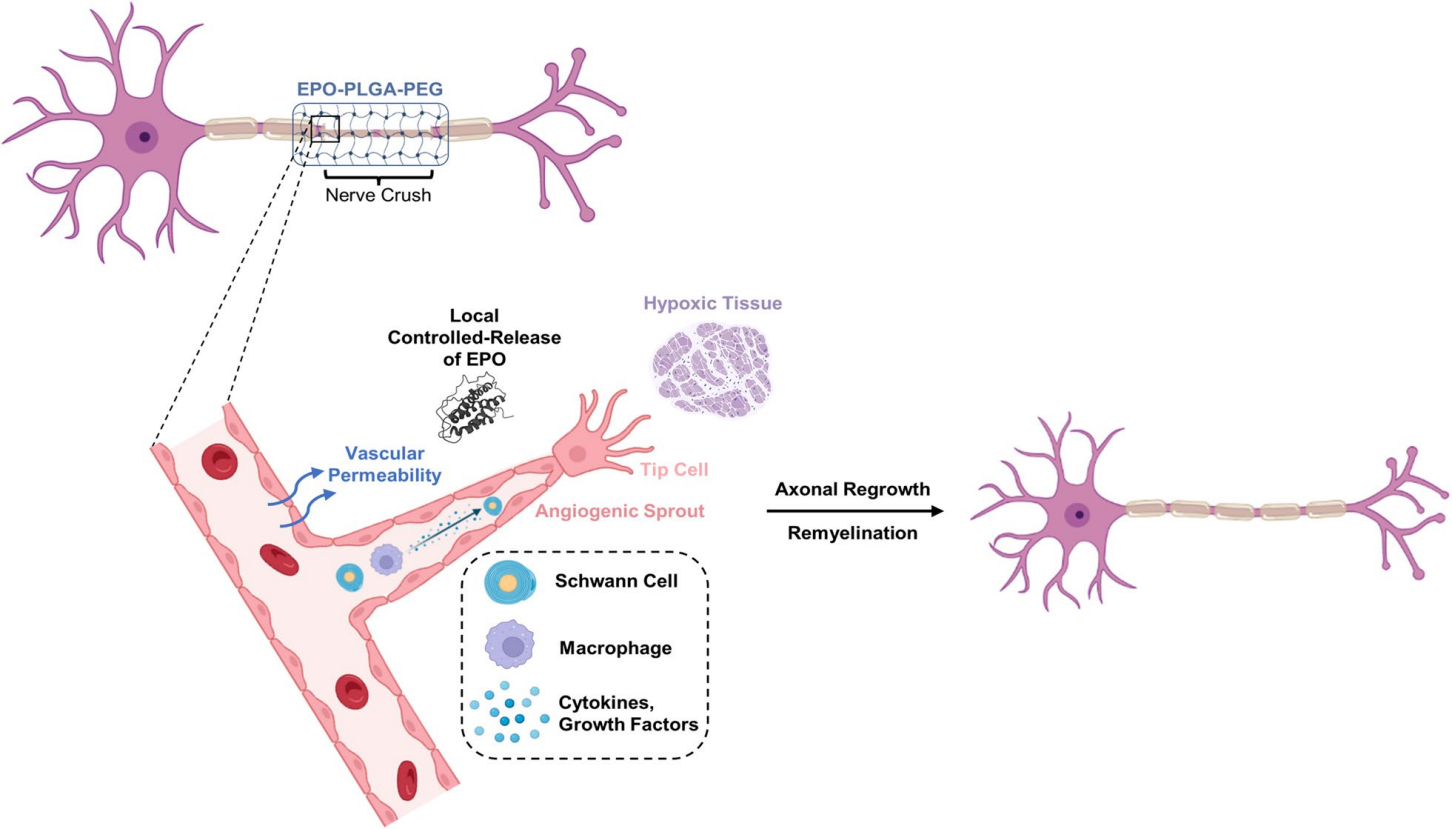
Schematic representation of an EPO-loaded thermogel as a local controlled-release delivery system

Block copolymers are biodegradable, thermosensitive drug delivery systems that allow for sustained drug release [20, 21]. We developed an EPO block copolymer consisting of amphiphilic polyethylene glycol/poly lactic acid-co-glycolic acid (PEG/PLGA) polymers. With a lower critical solution temperature around body temperature, we engineered EPO-PLGA-PEG, or EPO gel, so it can be injected as a liquid at room temperature and subsequently form an in situ gel around a nerve injury at body temperature for controlled-release of EPO. The topical effects of EPO at the site of a crush injury were unknown. We hypothesized that increasing the local concentration of this pro-angiogenic factor in a block copolymer would promote angiogenesis, nerve regeneration, and functional recovery (Fig. 1). Given the evidence that systemic EPO improves functional recovery after injury, we investigated whether releasing EPO at a controlled rate through a physical scaffold directly at a nerve injury could create a microenvironment more conducive to neurovascular regeneration and recovery.

## Results

### Block Copolymer synthesis and characterization

**Fig. 2:**
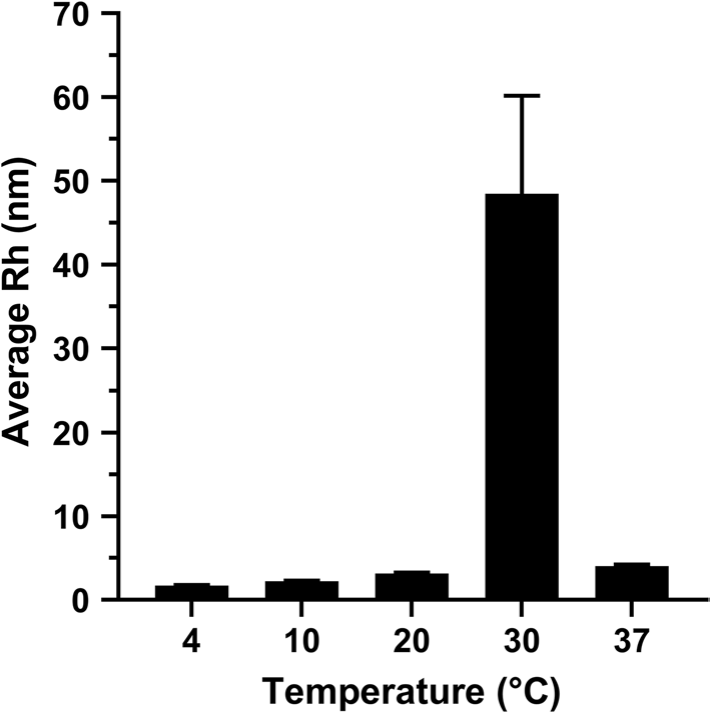
Dynamic light scattering of copolymer aqueous solutions. DLS measurements of EPO-PLGA-PEG polymer solutions showing the changes in hydrodynamic radius with temperature (n = 3/group; mean ± SEM).

To investigate the co-assembly behavior of PLGA-PEG polymers incorporated with EPO, we studied the sizes of micelles formed in dilute polymer solutions (0.1%) at varying temperatures (Fig. 2). At 4 °C, the initially formed micelle size was 1.7 nm (Table 1). No appreciable changes were observed in assembly size at temperatures 4 °C, 10 °C, and 20 °C. At 30 °C, significant increases in hydrodynamic radius and polydispersity were observed to 48.47 nm and 0.058, respectively (Table 1). Since the gelation process generates networks of polymer threads, the increases in particle size and polydispersity index (PDI) around gelation temperature provide a qualitative measure of assembly properties [21]. This suggests a transformation from micelle to gel network structure.

PDI is used to describe the degree of non-uniformity of particle size distribution, where a PDI of 0 suggests a perfectly uniform sample with respect to particle size and PDI values smaller than 0.05 are associated with highly monodisperse solutions [22]. At 4 °C, 10 °C, 20 °C, and 37 °C, PDI values were less than or equal to 0.002, suggesting highly monodisperse solutions. At 30 °C, PDI increased to 0.058, suggesting an increase in size heterogeneity due to the gelation process, but still representative of a monodisperse solution (Table 1).

**Table 1:** Table of DLS measurements including hydrodynamic radius (nm), molecular weight (MW, kD), and polydispersity index (PDI) at varying temperatures (n = 3/group; mean ± SEM).

Temperature (°C)	Hydrodynamic Radius (nm)	MW (kD)	PDI
4	1.7 ± 0.06	4.62	0.001
10	2.24 ± 0.08	7.45	0.001
20	3.15 ± 0.1	13.62	0.001
30	48.47 ± 11.67	1720.21	0.058
37	4.01 ± 0.2	20.93	0.002

### Rheological characterization of Block Copolymer Aqueous Solutions

**Fig. 3:**
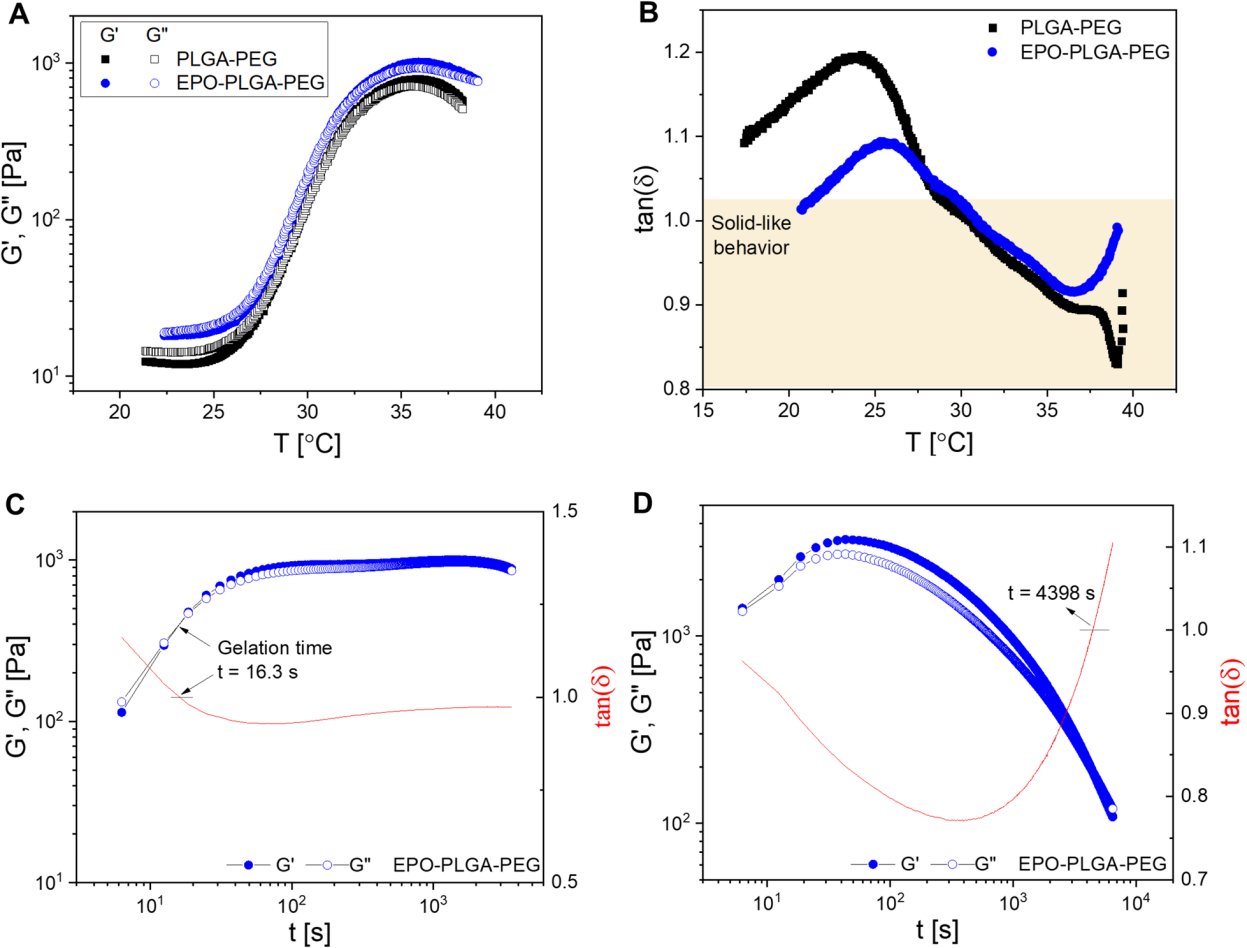
Rheological investigation of copolymer aqueous solutions. **A** Gelation temperatures of PLGA-PEG and 0.5 IU/µL EPO-PLGA-PEG were 30.4 and 30.9 °C, respectively (n = 3/group). **B** Tan(δ) was less than 1, indicating solid-like behavior for PLGA-PEG and EPO-PLGA-PEG in the physiologic temperature range (n = 3/group). **C** Solution to gel (sol-gel) transition of EPO-PLGA-PEG occurred in 16.3 s. Plateau of the moduli over time after gelation indicated the persistence of solid-like behavior (n = 3/group). **D** Sol-gel transition of EPO-PLGA-PEG was fully reversible and occurred in 4398 s (n = 3/group)

Oscillatory rheology was used to assess the suitability of our formation as a thermoresponsive in situ drug depot. Specifically, we monitored G’, the storage modulus which represents the elastic/solid-like property, and G’’, the loss modulus which represents the viscous/liquid-like property [23]. Both PLGA-PEG-PLGA and PLGA-PEG-PLGA polymers with EPO were soluble in PBS at room temperature and underwent solution-to-gel (sol-gel) transitions with increasing temperature. Figure 3A shows the change in modulus of PLGA-PEG and EPO-PLGA-PEG as a function of temperature. At low or room temperature, the loss modulus G’’ was greater than the storage modulus G’ reflecting a solution-free flowing phase. An abrupt increase in modulus was observed along with the formation of physical hydrogels as the temperature increased. The sol-gel transition temperatures (T_gel_), the crossover point of G’’ and G’, of PLGA-PEG and EPO-PLGA-PEG were 30.4 and 30.9 °C, respectively (Fig. 3A). In this way, the gel window of the EPO-PLGA-PEG covered body temperature, indicating that the formulation was suitable for our biomedical application. Similarly, the loss factor tan(δ) was less than 1 for PLGA-PEG and EPO-PLGA-PEG at temperatures at and above approximately 31 °C, indicating solid-like behavior and gel formation at physiologically relevant temperatures (Fig. 3B).

Figure 3C and D show the change in moduli as a function of time. As observed in Fig. 3C, sol-gel transition for EPO-PLGA-PEG occurred rapidly within 16.3 s. Plateau of the moduli over time indicated persistence of solid-like behavior and stability of the formed physical gel. After gelation, the storage modulus of PLGA-PEG and EPO-PLGA-PEG stabilized at around 820 and 980 Pa, respectively. This change in magnitude of the moduli indicates that EPO-PLGA-PEG is a stronger gel than vehicle alone. The dynamic time sweep performed in Fig. 3D, ramping down from high to low temperature, was employed to study reversibility of gelation. We found that the sol-gel transition was fully reversible and occurred in 4398 s.

### Release kinetics and Biophysical characterization of EPO from Hydrogels

**Fig. 4:**
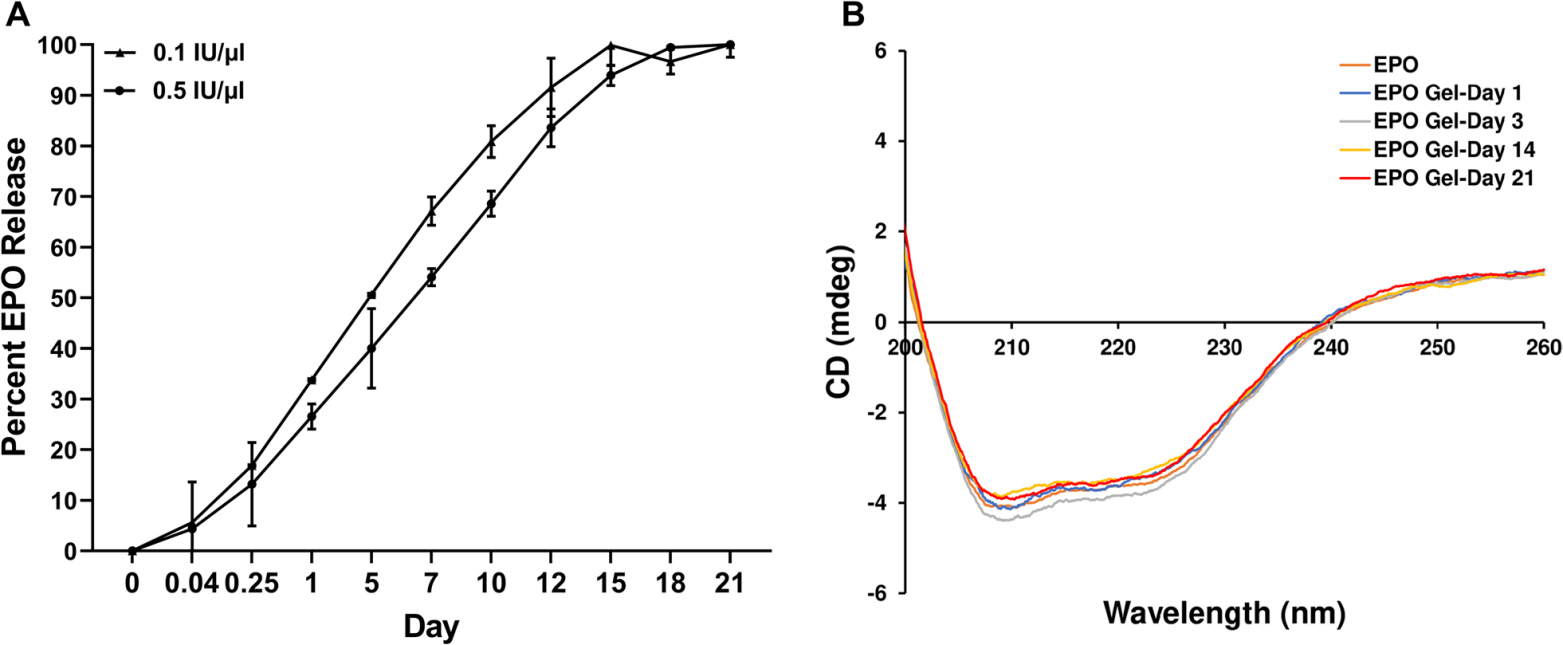
Release kinetics and CD spectroscopy of EPO-PLGA-PEG. **A** Percent of total encapsulated EPO from PLGA-PEG hydrogels (n = 3/group; mean ± SEM). **B** CD spectroscopy of released EPO at different time intervals from PLGA-PEG hydrogels (n = 3/group)

We evaluated the in vitro release profiles of EPO from PLGA-PEG-PLGA at varying concentrations (0.1 IU/µL, 0.5 IU/µL) to determine if the thermogel could release clinically relevant doses of EPO at a controlled rate. The data in Fig. 4A show the cumulative amounts of EPO released over 21 days. Initially, on the first day, approximately 33.8 and 26.5% were released for 0.1 IU/µL and 0.5 IU/µL, respectively (Fig. 4A). This uncontrolled release behavior can be attributed to loosely bound EPO molecules located at the vicinity of the hydrogel − solution interface. In the second phase of release, the release of EPO was more sustained. 0.5 IU/µL solutions released an additional 27.6% of cargo molecules from day 1 to day 7 compared to 33.3% additional release for 0.1 IU/µL (Fig. 4 A). During this phase, EPO is released through not only diffusion like during burst release, but hydrolytic degradation of PLGA. By day 12, 91.6% and 83.6% were released for 0.1 IU/µL and 0.5 IU/µL, respectively. The slower release of EPO from 0.5 IU/µL may be due to local aggregation and precipitation which imparts greater resistance to degradation compared to a lower protein concentration [21]. For this system, EPO release was completed after 18 days for both concentrations and cumulative release was proportional to the total loaded amount of EPO. We chose 0.5 IU/µL EPO concentration for animal experiments as it demonstrated a less aggressive initial burst release and more controlled delivery over time.

To determine if released EPO was conformationally stable, we evaluated structural stability with circular dichroism (CD) spectroscopy over the release course. Figure 4B shows the CD spectra of native EPO which are typical of alpha-helical protein, with negative bands at 208 and 222 nm regions [24, 25]. No significant change of EPO conformation was observed even after 21 days of release (Fig. 4B). This suggests that the majority of encapsulated EPO maintains its secondary conformation in comparison to native EPO.

### In vivo degradation of EPO-PLGA-PEG and EPO Release in mice

**Fig. 5:**
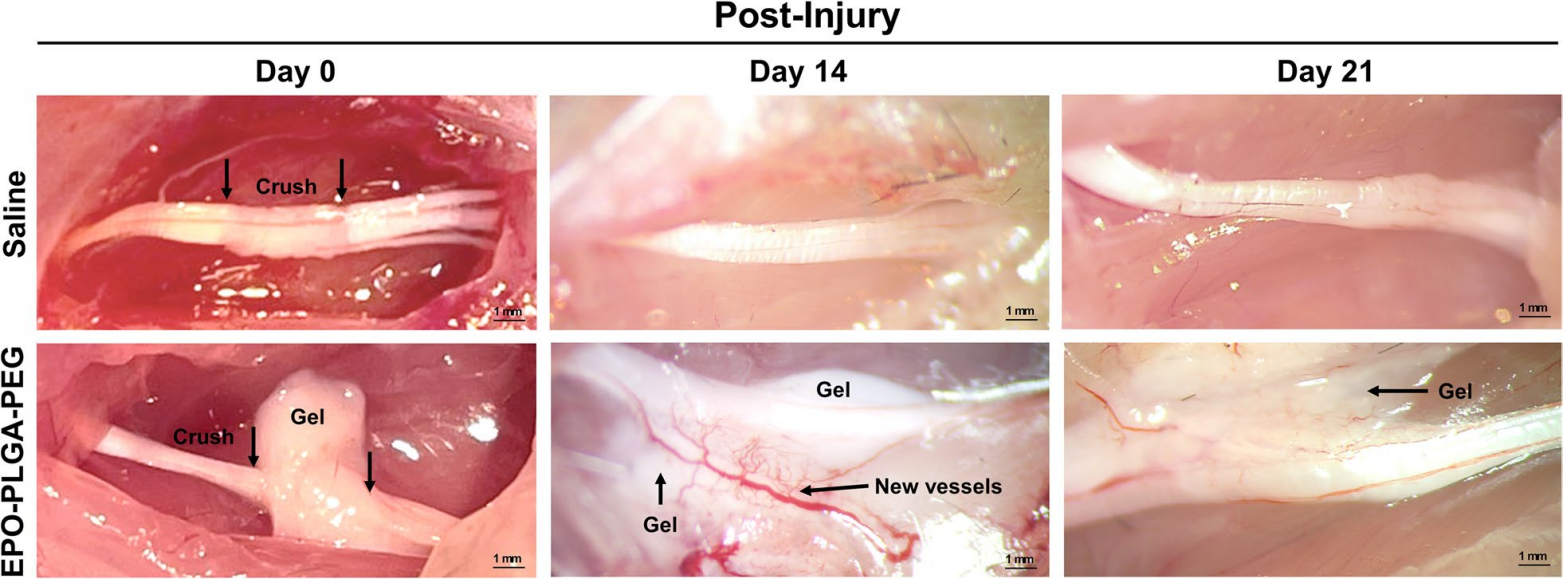
Representative images of in vivo biodegradation of EPO-PLGA-PEG after post-crush injury administration. EPO-PLGA-PEG was administered directly onto the 3 mm sciatic nerve crush site, as shown by the arrows, on day 0. On post-injury day 14, blood vessels were abundant at the injection and peri-injection site post-injury, forming a microvascular network. The gel was still present on the nerve on day 21 (n = 5/group; scale bar = 1 mm)

To assess the suitability of EPO-PLGA-PEG as an in situ drug depot, we surgically exposed the injured sciatic nerve to observe the gel location, adherence, integrity, and local effects after administration. As shown in Fig. 5, the 3 mm crush injury was clearly visible after injury. Immediately after injury, we administered EPO-PLGA-PEG onto the injury site. By day 14, blood vessels were abundant at the injection site, forming a microvascular network as shown by the arrows (Fig. 5). In contrast, most tissue in saline and vehicle-treated animals had little evidence of blood vessel formation at or around the injury site. In addition, the gel was still present on the nerve on day 21, demonstrating its physical adherence to the tissue as well as controlled degradation rate.

**Fig. 6:**
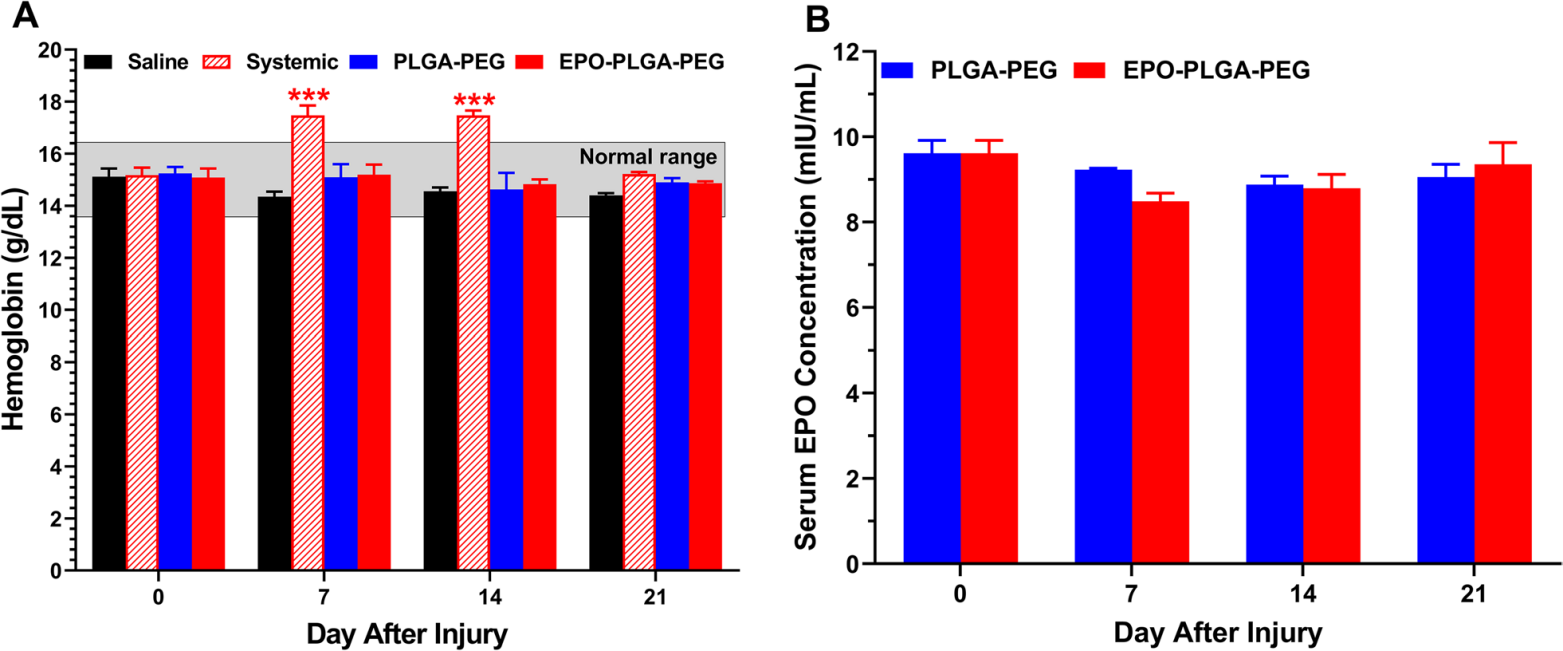
Effects of EPO-PLGA-PEG on hemoglobin and in vivo release in mice. **A** EPO-PLGA-PEG was not associated with a significant increase in hemoglobin whereas systemic EPO resulted in a significant increase on days 7 and 14 outside of the normal range (n = 5/group; mean ± SEM; ^***^p < 0.001 vs. saline, PLGA-PEG, and EPO-PLGA-PEG groups). **B** EPO-PLGA-PEG was not associated with a significant increase in serum EPO (n = 5/group; mean ± SEM).

Burst release in drug delivery systems can pose a safety concern if the drug concentration reaches beyond the toxicity threshold [26]. Since sustained elevated concentrations of EPO can cause increased red blood cell production; we evaluated the effect of our local EPO formulation on hemoglobin levels [18]. For C57BL/6 J mice of this age and sex, the normal hemoglobin range is 13.6 to 16.4 g/dL [27]. EPO-PLGA-PEG was not associated with a significant increase in hemoglobin levels at any timepoint (Fig. 6A). On the other hand, systemic EPO administration resulted in a significant increase in hemoglobin outside of the normal physiologic range on days 7 and 14 (Fig. 6 A). In addition, we investigated the effect of EPO-PLGA-PEG on serum EPO concentration, in which the normal human range has been shown to be 5.8–9.9 IU/mL in males [27]. Based on this range, EPO-PLGA-PEG did not result in a significant increase in serum EPO (Fig. 6B).

### EPO-PLGA-PEG effect on neuromuscular functional recovery

**Fig. 7:**
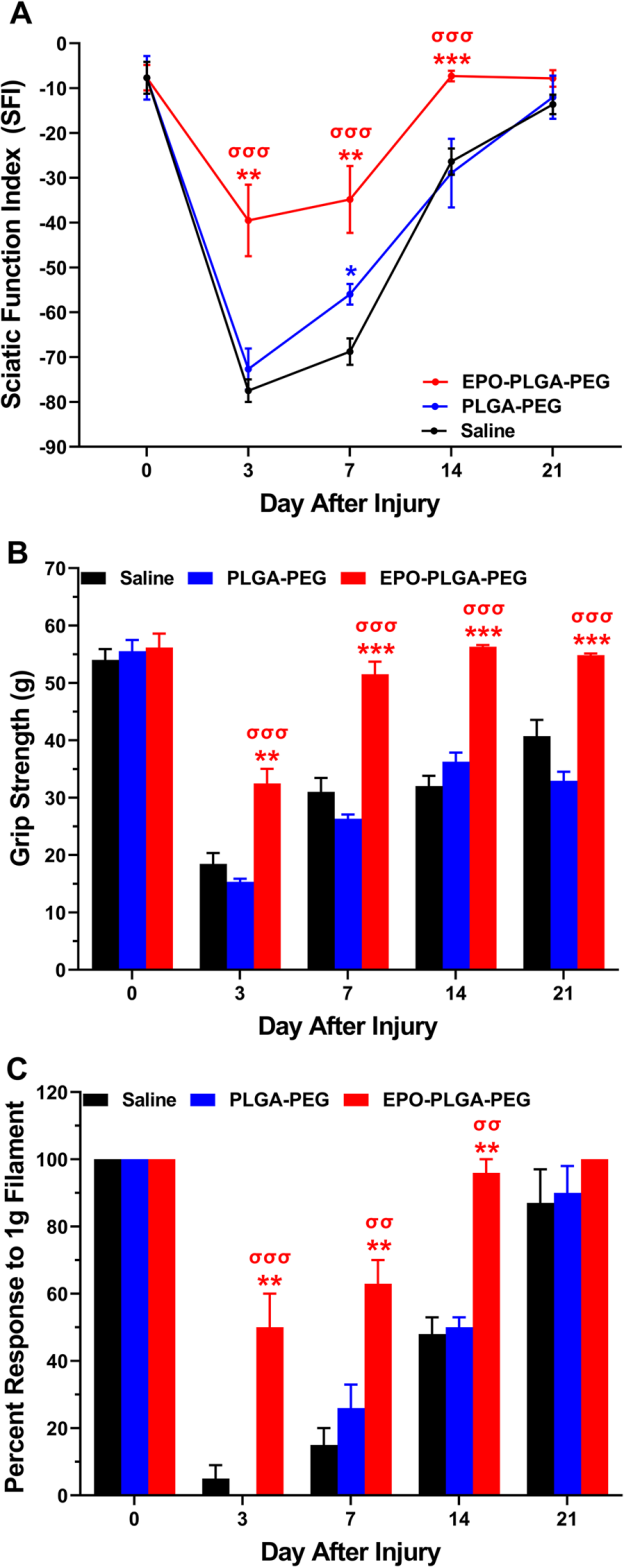
Effects of EPO-PLGA-PEG on motor and sensory functional outcomes post-crush injury. **A** EPO-PLGA-PEG significantly improved SFI on days 3, 7, and 14 compared to saline and vehicle groups. **B** EPO-PLGA-PEG significantly improved grip strength on days 3, 7, 14, and 21 post-injury compared to saline. **C** EPO-PLGA-PEG significantly improved withdrawal reflex (percent response to filament) as compared to saline on post-injury days 3, 7, and 14 (n = 5/group; mean ± SEM; ^*^p < 0.05, ^**^p < 0.01, and ^***^p < 0.001 vs. saline group; ^σσ^*p* < 0.01 and ^σσσ^*p* < 0.001 vs. PLGA-PEG group)

In previous studies, we have shown that systemic EPO administration improves motor and sensory functional recovery after sciatic nerve crush injury [17, 18]. The effects of a local thermoresponsive formulation of EPO on motor and sensory outcomes post-peripheral nerve injury were unknown. We found EPO-PLGA-PEG improved post-injury SFI on days 3 (− 39.5 vs. − 77.5), 7 (− 34.8 vs. − 68.8), and 14 (− 7.3 vs. − 26.4) compared to saline (Fig. 7A, ^**^p < 0.01, ^***^p < 0.001). EPO-PLGA-PEG also significantly improved SFI on days 3, 7, and 14 compared to PLGA-PEG (Fig. 7A, ^σσσ^*p* < 0.001).

To further evaluate motor recovery, we studied post-injury grip strength. EPO-PLGA-PEG significantly improved grip strength on post-injury days 3 (32.5 g vs. 18.5 g), 7 (51.5 g vs. 31 g), 14 (56.3 g vs. 32 g), and 21 (54.8 g vs. 40.7 g) compared to saline (Fig. 7B,^**^p < 0.01, ^***^p < 0.001). EPO-PLGA-PEG also significantly improved grip strength on days 3, 7, 14, and 21 compared to PLGA-PEG (Fig. 7B, ^σσσ^*p* < 0.001). Taken with SFI data, EPO-PLGA-PEG treatment improves volitional muscle strength in proportion to improved global motor function after nerve injury.

To assess the effect of EPO-PLGA-PEG on sensory nerve recovery, we performed von Frey filament testing. In addition to improving motor outcomes, EPO-PLGA-PEG treatment significantly improved withdrawal reflex (percent response to filament) as compared to the saline group on post-injury days 3 (48% vs. 4%), 7 (64% vs. 16%), and 14 (96% vs. 48%) (Fig. 7C, ^**^p < 0.01). EPO-PLGA-PEG also significantly improved withdrawal reflex on days 3, 7, and 14 compared to PLGA-PEG (Fig. 7C, ^σσ^*p* < 0.01, ^σσσ^*p* < 0.001). These findings demonstrate that EPO-PLGA-PEG can improve sensory and motor nerve function after crush injury.

### EPO-PLGA-PEG effect on neurovascular regeneration and remyelination

**Fig. 8:**
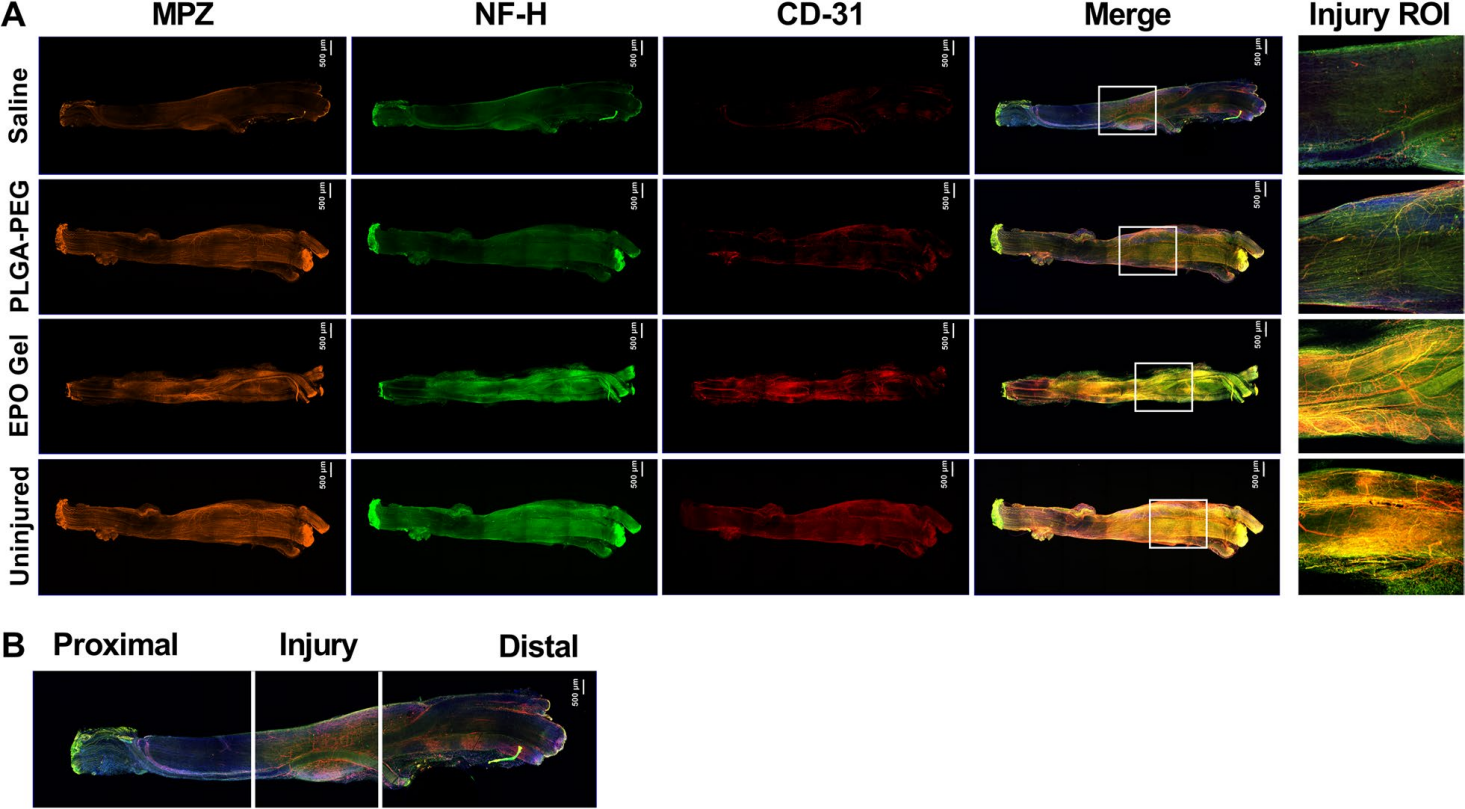
Evaluation of whole-mount immunostaining of the crushed nerve on post-injury day 21 for nerve fiber, myelin, and blood vessel distribution. **A** Representative compact images of immunofluorescence staining of the whole nerve for MPZ in orange, NF-H in green, CD31 in red, merged view, and region of interest (ROI) in the injury zone. **B** For the purpose of evaluation and quantification, each imaged whole nerve was divided into 3 zones: proximal, injury, and distal. The white box represents the injury ROI (n = 5/group; magnification = 5x; scale bar = 500 μm). Each image represents 3 images from 5 different SNs for a total of 15 images per group

To evaluate the distribution and alignment of regenerating nerve filaments and blood vessels, immunofluorescence staining of the whole nerve was performed for neurofilament-H (NF-H), myelin protein zero (MPZ), and CD31, a marker for angiogenesis (Fig. 8A). On day 21 post-injury, all nerves were found in good continuity. To investigate the effect of EPO-PLGA-PEG on angiogenesis, AngioTool was used to measure blood vessel characteristics in CD31-stained whole nerves. Each nerve was divided into proximal, injury, and distal zones to quantify the distribution in each region, since Wallerian degeneration causes specific changes distal to nerve injury (Fig. 8B).

**Fig. 9:**
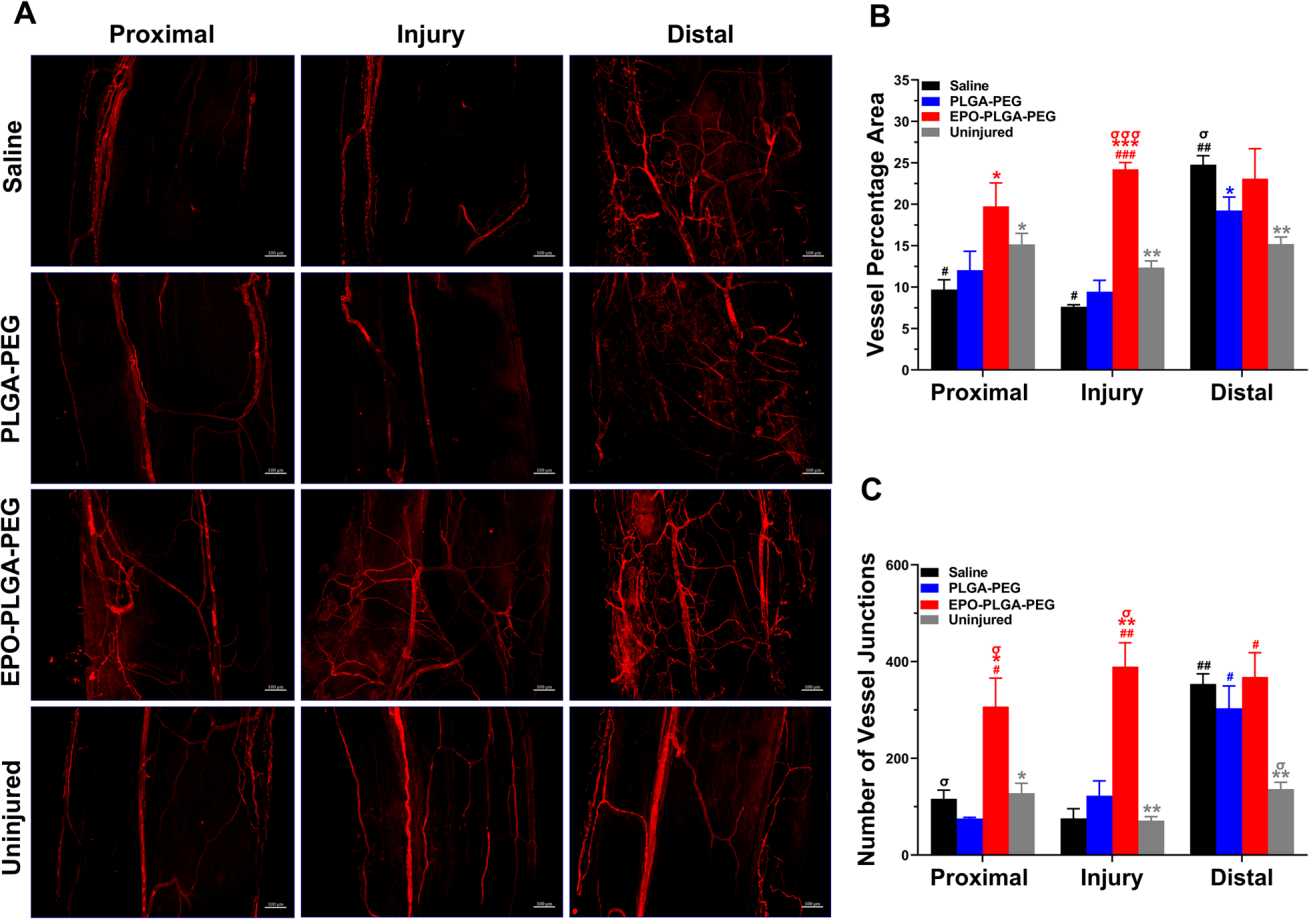
Effects of EPO-PLGA-PEG on whole-mount nerve angiogenesis post-crush injury. **A** Representative CD31-stained whole-mount images to show blood vessel formation. **B** Blood vessel density is shown as the percentage of number of blood vessels in total area. **C** Branching of blood vessels is shown as the number of blood vessel junctions/mm^2^ (n = 5/group; mean ± SEM; ^*^p < 0.05, ^**^p < 0.01, and ^***^p < 0.001 vs. saline group; ^σ^*p* < 0.05 and ^σσσ^*p* < 0.001 vs. PLGA-PEG group; ^#^*p* < 0.05, ^##^*p* < 0.01, and ^###^*p* < 0.001 vs. uninjured group; scale bar = 100 μm). Each image represents 3 images from 5 different SNs, for a total of 15 images analyzed per group

Angiogenesis is one of the most crucial initial processes after nerve injury, playing an essential role in axonal sprouting, regeneration, and reinnervation [28, 29]. Macroscopically, the microvessel density was significantly higher in the EPO-PLGA-PEG group which indicated an optimal regenerated condition for tissue recovery (Fig. 9A). In the uninjured nerves, CD31 staining was uniform over the entire length. EPO-PLGA-PEG nerves also had uniform staining across all three zones. In contrast, in saline-treated injured nerves, CD31 staining intensities in the injury and distal zones were less pronounced compared to the proximal zone.

AngioTool images clearly depicted the blood vessel architecture (red lines) and their branching points (blue dots) used for quantification (Additional file 1: Fig. S2). Quantification of vessel percentage area, or vessel density, revealed that the EPO-PLGA-PEG group had a higher blood vessel density in the injury and proximal zones compared to saline (Fig. 9B). All groups had an increased vessel density in the distal zone compared to uninjured control. Similarly, the number of vessel junctions was significantly increased in all groups in the distal zone compared to uninjured. However, EPO-PLGA-PEG resulted in a significant increase in number of junctions in the proximal and injury zones as well. This effect was significant compared to vehicle and saline treatments. Of note, blood vessel density and branching index levels in the EPO-PLGA-PEG group were relatively comparable between all three zones, indicating an enhanced directional angiogenesis.

**Fig. 10:**
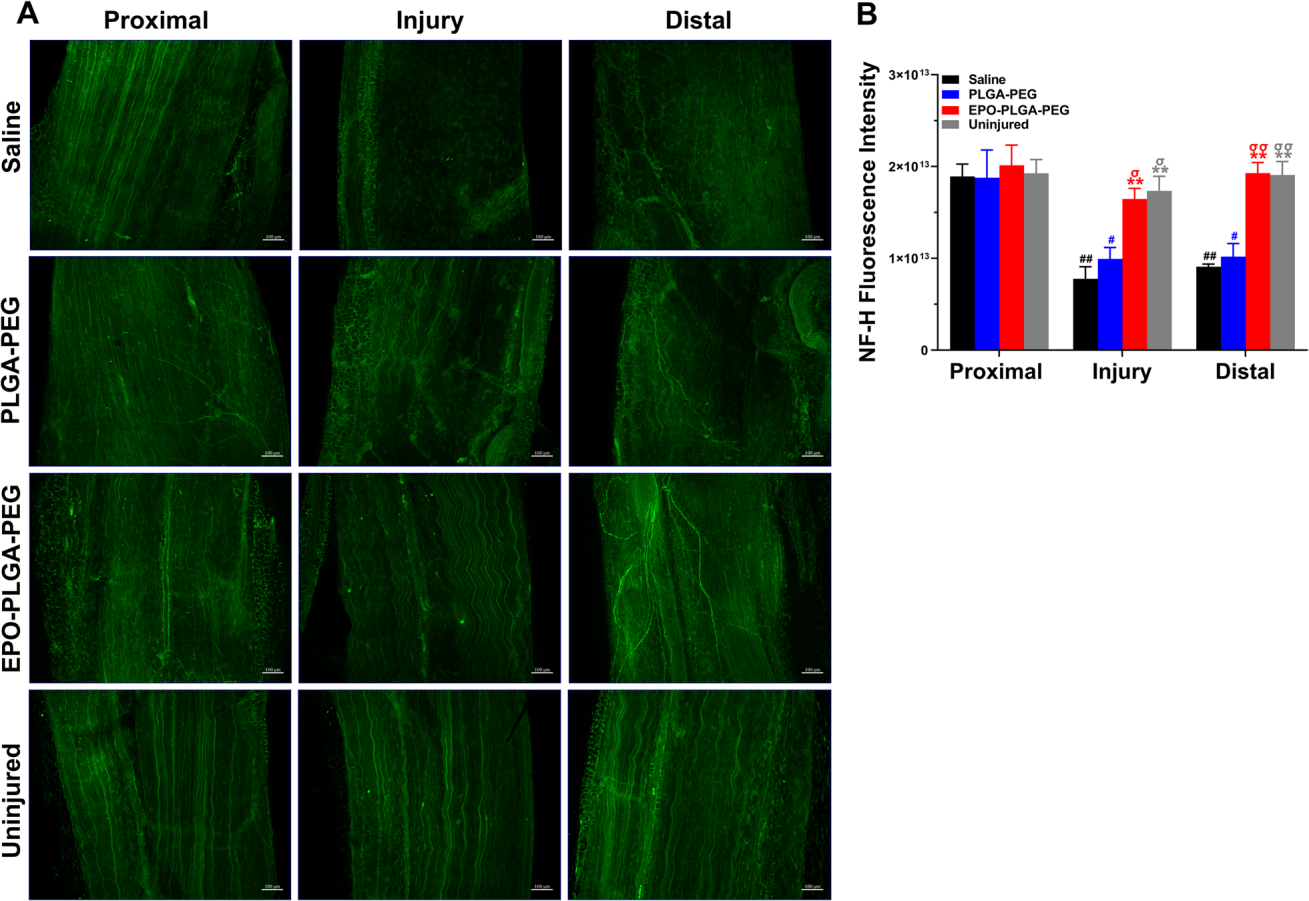
Effect of EPO-PLGA-PEG on whole-mount nerve fiber distribution post-crush injury. **A** Representative NF-H-stained whole-mount images to display nerve fibers. **B** Quantification of NF-H integrated density (n = 5/group; mean ± SEM; ^**^p < 0.01 vs. saline group; ^σ^*p* < 0.05 and ^σσ^*p* < 0.01 vs. PLGA-PEG group; ^#^*p* < 0.05 and ^##^*p* < 0.01 vs. uninjured group; scale bar = 100 μm). Each image represents 3 images from 5 different SNs, for a total of 15 images analyzed per group

**Fig. 11:**
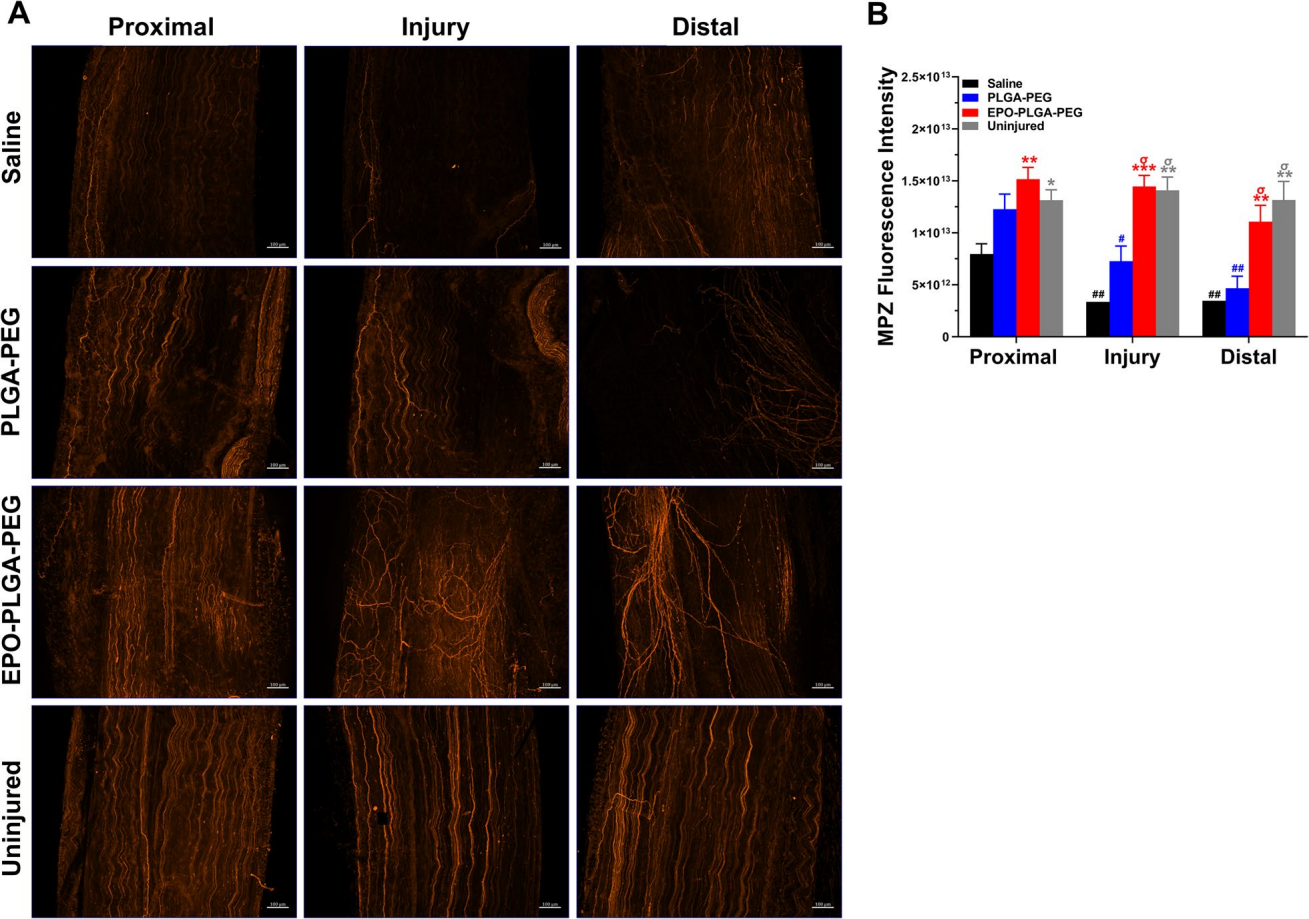
Effect of EPO-PLGA-PEG on whole-mount nerve myelin distribution post-crush injury. **A** Representative MPZ-stained whole-mount images. **B** Quantification of MPZ integrated density (n = 5/group; mean ± SEM; ^*^p < 0.05, ^**^p < 0.01, and ^***^p < 0.001 vs. saline group; ^σ^*p* < 0.05, ^σσ^*p* < 0.01, and ^σσσ^*p* < 0.001 vs. PLGA-PEG group; ^#^*p* < 0.05, ^##^*p* < 0.01, and ^###^*p* < 0.001 vs. uninjured group; scale bar = 100 μm). Each image represents 3 images from 5 different SNs, for a total of 15 images analyzed per group

Since new blood vessel formation is vital for nerve regeneration, we hypothesized that increasing the local concentration of pro-angiogenic EPO at an injury would lead to enhanced nerve regeneration and remyelination [12, 13]. Using whole-mount staining, the pattern of axonal re-growth can be clearly revealed following injury. Figures 10 and 11 show representative images of NF-H and MPZ-stained whole-mount nerves, respectively. Uninjured nerves displayed normal nerve architecture with uniform NF-H (Fig. 10A) and MPZ (Fig. 11A) staining over the entire length. Unidirectional nerve fibers were compactly packed and parallelly aligned and had comparable NF-H intensity across all three regions. In contrast, NF-H staining in the injury and distal zones of saline group revealed significantly less neurofilaments. This is due to Wallerian degeneration which causes axonal and myelin sheath disintegration distal to nerve injury [30]. However, EPO-PLGA-PEG treatment resulted in significantly more nerve fibers in the injury and distal regions compared to saline and vehicle treatments. Comparable levels of nerve fibers across the length of EPO-PLGA-PEG nerves suggest a more complete regeneration process compared to other groups. As compared to other groups, we also observed greater numbers of compactly packed, parallelly aligned myelinated axons in both injury and distal zones of EPO-PLGA-PEG group. As expected, regenerated axons in all injured groups were less straight than the uninjured group.

Myelination is one of the main features contributing to nerve conduction and the extent of myelination correlates with functional recovery [31]. Compared to all groups, MPZ staining in the proximal zone of the saline group revealed significantly less myelin. EPO-PLGA-PEG treatment resulted in significantly higher myelin counts in the injury and distal regions compared to saline and vehicle treatments. Comparable levels of myelin across the length of EPO-PLGA-PEG nerves suggest a more complete myelination process, resembling the myelin distribution of uninjured nerves.

**Fig. 12:**
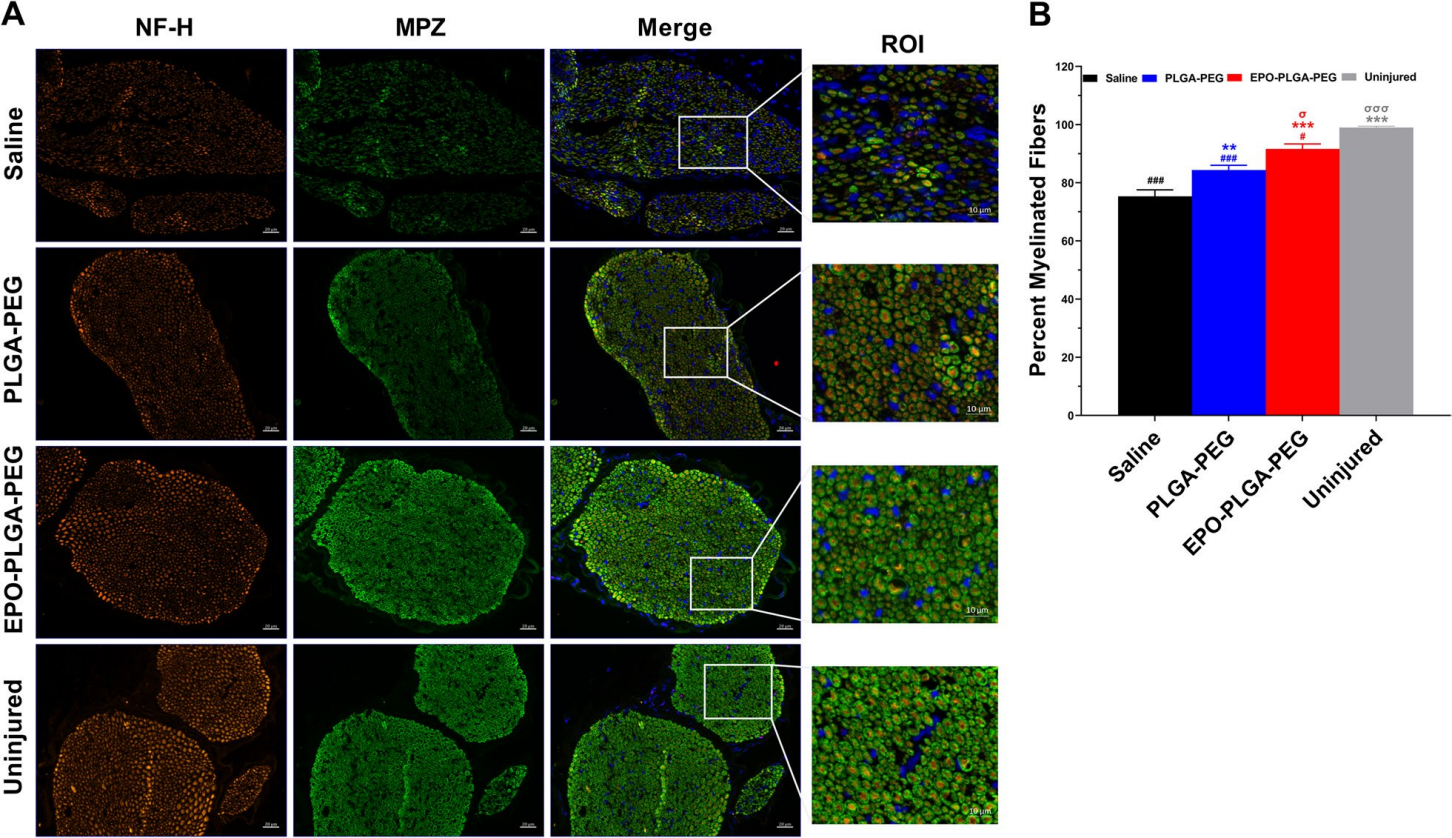
Effect of EPO-PLGA-PEG on axon morphology and myelinated fibers. **A** Representative transverse sciatic nerve immunofluorescent images of nuclei (DAPI), NF-H, and MPZ sectioned at the level of injury on day 21. **B** Quantification of percent myelinated fibers (n = 5/group; mean ± SEM; ^**^p < 0.01 and ^***^p < 0.001 vs. saline group; ^σ^*p* < 0.05 and ^σσσ^*p* < 0.001 vs. PLGA-PEG group; ^#^*p* < 0.05 and ^###^*p* < 0.001 vs. uninjured group; scale bar = 10 μm). Each image represents 3 images from 5 different SNs, for a total of 15 images analyzed per group

The enhancement of functional recovery at early timepoints was too rapid to be attributed to axonal regeneration alone. We therefore tested the hypothesis that EPO-PLGA-PEG preserves or restores myelination on spared axons. To determine the effect of EPO-PLGA-PEG on nerve remyelination, transverse sectioned nerves cut at the level of the crush injury were stained for NF-H and MPZ using immunohistochemistry to quantify percent of myelinated fibers [32, 33]. There was a visually marked decrease in peripheral myelin at the site of crush injury for both saline and PLGA-PEG-treated nerves (Fig. 12A). The injury region of interest for EPO-PLGA-PEG closely resembled that of uninjured, with tightly packed myelinated axons. In contrast, the saline group ROI demonstrated fewer myelinated axons which were more heterogenous and contained more DAPI-positive inflammatory infiltrates (Fig. 12A). In saline mice, only 75.3 +/− 2.2% of surviving axons (defined by neurofilament expression) were myelinated, while 91.7 +/− 1.7% of the axons were myelinated in mice treated with EPO-PLGA-PEG (^***^p < 0.001). EPO-PLGA-PEG resulted in a significantly higher percent of myelinated fibers compared to both saline (^***^p < 0.001) and vehicle (^σ^*p* < 0.05) groups.

## Discussion

This study used PLGA-PEG-PLGA block copolymers to encapsulate the pro-angiogenic protein EPO in FDA-approved, biodegradable, and biocompatible hydrogels. We anticipated that a block copolymer formulation could be used as a versatile vector for the delivery of protein to a localized nerve injury [20]. We engineered a novel thermoresponsive formulation of EPO which undergoes a liquid-to-solid phase transition at physiologically relevant temperatures, making it an ideal candidate for in situ controlled-release drug delivery. We demonstrate that sustained local delivery of EPO at a nerve crush site stimulates new blood vessel formation, axonal growth, and remyelination post-injury. These processes were associated with enhanced recovery of neuromuscular function. Overall, these findings suggest that local delivery of EPO via PLGA-PEG thermogelling polymers may effectively promote neurovascular regeneration and functional recovery following PNI.

Our previous studies demonstrated the efficacy of systemic EPO administration in promoting functional recovery and mitigating inflammatory, angiogenesis, and myelination components of nerve injury [16, 18]. However, systemic administration requires patient compliance and is contraindicated in patients with certain underlying conditions. In patients who are candidates for systemic treatment, hematologic monitoring is essential as EPO causes increases in red blood cell count and hemoglobin levels, increasing the risk of adverse side effects [34]. Unlike systemic EPO, EPO-PLGA-PEG was not associated with a significant increase in serum EPO concentration and therefore had no significant effect on blood parameters such as hemoglobin. This is due to local delivery of EPO directly at the site of injury where perineurial and blood-nerve (endoneurial) barriers are breached. This provides the opportunity for direct placement of EPO-PLGA-PEG into the peripheral nerve environment to help bypass the blood-nerve barrier and other drug metabolism barriers presented with systemic administration [35, 36]. Another benefit of this delivery system is the ability to maintain high local concentrations directly at the injury site for approximately 18 days with a single administration, as EPO given systemically has a relatively short half-life in serum [37]. Overall, these results may indicate a decreased need for hematologic monitoring and less potential for adverse side effects, providing clinical advantages over systemic treatment.

Controlled-release delivery of proteins can be difficult due in part to the harsh chemical environment and sensitivity of proteins to temperature, pH, and salts [38]. Because of this, it is important to consider the molecular interactions between the protein and polymer matrix since too little attraction will hinder controlled-release and too strong attraction will cause the protein to remain in the gel matrix. Benefits of this hydrogel-based system include limited molecular motions and PLGA’s hydrophilic environment which allow EPO to remain stable and protect it from enzymatic degradation. Because of block copolymers’ balance of hydrophobic and hydrophilic units, our in vitro release studies indicated an appropriate interaction between EPO and PLGA-PEG micelles. Moreover, CD spectra indicated that released EPO was conformationally stable throughout the release course, a crucial aspect for controlled-release protein delivery. This may be attributed to PEG’s ability to extend EPO half-life and direct PEGylation conferring benefits in both protein absorption and systemic stability [39, 40].

There are previously published biomaterials for nerve regeneration, including nerve conduits and hydrogel delivery systems. Vascularized nerve conduits are currently being explored with the goal of combining surgical nerve repair with vascularization. However, conduits must be surgically placed and have time and cost limitations [41]. Unlike nerve conduits, EPO-PLGA-PEG can be locally injected into an injured limb, reducing the need for surgical exposure of the nerve [20]. In addition to nerve conduits, natural hydrogel systems such as hyaluronic acid, chitosan, and collagen have been published for peripheral nerve regeneration [41–43]. Unlike synthetic PLGA-PEG, these biomaterials have poor mechanical properties and not as easily tunable degradation profiles [41].

In this study, we found that EPO-PLGA-PEG enhanced both the speed and extent of motor and sensory functional recovery after PNI, as evidenced by SFI, grip strength, and von Frey measurements. We hypothesized that our formulation would result in greater functional improvements than systemic treatment by allowing us to precisely deliver higher protein concentrations directly to the site of injury. We observed the steepest SFI improvement from EPO-PLGA-PEG on day 3 post-injury, an earlier timepoint than systemic treatment in previous studies. This is likely related to the high burst release of EPO within the first day of the release course, as functional improvements are expected to follow peaks in drug concentration.

A favorable vascular microenvironment is crucial to recovery following PNI [44]. After peripheral nerve injury, rapid vascular network reconstruction is a prerequisite for nerve regeneration and function restoration [39]. For example, local delivery of VEGF was found to promote the invasion of Schwann cells and neovascularization after nerve injury, indicating that both endogenous and exogenous administration of angiogenesis factors are vital for nerve regeneration [45]. Based on previous systemic EPO findings, we hypothesized that sustained delivery of pro-angiogenic EPO would facilitate angiogenesis and axonal regrowth, increasing blood vessel density at the site of nerve injury. EPOR is also preferentially expressed on peripheral nerves, further validating our rationale for local delivery [30]. We show in the current study that enhanced angiogenesis is associated with improved neurogenesis at the injury site. In particular, we observed increased regenerated and myelinated nerve fibers in regions closely associated with newly formed blood vessels. Our findings are consistent with other studies showing that use of local angiogenic factors enhances angiogenesis, priming an injury for axonal regeneration [45, 46].

Despite the interesting findings with EPO-PLGA-PEG administration, our study has some limitations. First, we did not perform size exclusion chromatography to investigate the potential cleavage of EPO. Second, we studied tissue effects at one post-injury timepoint. Finally, we did not study the effects of our therapeutic on muscle morphology.

In summary, this study was particularly designed to characterize and determine the efficacy of EPO-PLGA-PEG in PNI treatment. In vitro characterization demonstrated that EPO-PLGA-PEG releases conformationally stable EPO at a controlled rate for several weeks. Rheological investigation showed that the formulation forms an in situ gel rapidly at body temperature, offering promise as a long-acting injectable therapeutic. Moreover, EPO-PLGA-PEG enhanced functional recovery, increased blood vessel density and branching, and promoted nerve regeneration and remyelination after PNI. Our findings have significant clinical implications for the use of EPO-PLGA-PEG as a locally injectable, long-acting treatment for PNI, reducing the need for frequent dosing and hematologic monitoring. Moreover, translation could be rapid as this delivery system is made of entirely FDA-approved components, fulfilling a significant clinical need for a disease in which no standard drug treatment currently exists.

## Conclusion

We have encapsulated the pro-angiogenic FDA-approved hormone EPO into biodegradable and biocompatible PLGA-PEG-PLGA triblock copolymers. This thermogel delivery system achieved sustained release of EPO at the injury epicenter for approximately 3 weeks and resulted in no adverse effects on hematologic parameters. EPO-PLGA-PEG promoted functional recovery and stimulated angiogenesis and neurogenesis after peripheral nerve crush injury in mice. This pre-clinical study demonstrates the applicability and efficacy of this minimally invasive therapy for a disease in which no universal pharmacologic agent currently exists.

## Methods

### Animals

Ten-week-old male C57BL/6 J mice (Jackson Laboratories, Bar Harbor, Maine) weighing 25 ± 3 g were used in this study. Experimental design and animal protocols were approved by the Institutional Animal Care and Use Committee (IACUC) at The Pennsylvania State University College of Medicine.

### Block Copolymer synthesis and characterization

Poly(lactide-co-glycolide)-b-Poly(ethylene glycol)-b-Poly(lactide-co-glycolide) (1700-1500-1700 Da, LA:GA 15:1, 94%/6% LA/GA, PolySciTech) and EPO (Epoetin alfa, PROCRIT) were used without further purification. EPO was incorporated in PLGA-PEG-PLGA triblock copolymer solution (1X PBS (phosphate-buffered saline), pH 7.4, polymer concentration: 20 wt%) and stirred at 4 °C until it was completely dissolved.

The hydrodynamic radius of the block copolymer solutions was evaluated by dynamic light scattering (DLS) using a Viscotek 802 DLS (Malvern) equipped with a 60 mW laser. A 15 µL sample diluted to 0.1% concentration (pH 7.4) was loaded into a quartz cell and the scattered light intensity was detected at 90° for 10 repeat runs. The samples were incubated at desired preset temperatures (4, 10, 20, 30, and 37 °C) in the DLS instrument before taking measurements. The hydrodynamic radius was evaluated from the averaged signal by the OmniSIZE software (Cumulants method) based on the measured values of the diffusion coefficient using the Stokes-Einstein equation.

### Quantification of EPO release from hydrogels

Polymer solutions containing varying concentrations of EPO (0.1 IU/µL, 0.5 IU/µL) were transferred to 1.5 mL microcentrifuge tubes (02-681-5, Fisher Scientific) and the samples were incubated in a water bath at 37 °C to convert them to physical hydrogels. Next, 1 mL of PBS (1X, pH 7.4) was added to each test tube as release media and the samples were left in the water bath at 37 °C for 21 days. At designated timepoints, 10 µL of release media was extracted from the tubes and replaced with the same amount of fresh PBS to maintain the sink condition [20]. The samples were stored at -20 °C until analyzed. The amount of EPO released into the media was measured with an enzyme-linked immunosorbent assay (BMS2035-2, Human EPO ELISA Kit, Thermo Fisher Scientific) to determine cumulative drug release over time. Quantification of amount of EPO released was done using a standard curve generated using EPO standards (Additional file 1: Fig. S1).

### Biophysical characterization of released EPO

The stability of protein cargoes released from PLGA-PEG block copolymer solutions was studied at different timepoints using CD spectroscopy. CD measurements were performed on a JASCO J-1500 spectrometer, equipped with a Peltier model PTC-517 thermostat cell holder. Signals were recorded from 260 nm to 180 nm with a scan speed of 50 nm/min and a band width of 1 nm at 20 ºC. The quartz cell used was 1 mm. The EPO release samples were prepared the same way as in ELISA experiments to ensure the final EPO concentration at each timepoint was comparable to in vitro release data. The measurements were taken in triplicate and the average values were plotted as mean residue ellipticity [21].

### Rheological characterization of Block Copolymer Aqueous Solutions

To investigate the suitability of our formulation as a thermoresponsive in situ drug depot, we studied gelation behavior using oscillatory rheology. Small amplitude oscillatory shear experiments were performed in a Discovery Hybrid Rheometer (DHR-3, TA Instruments, New Castle, DE, USA). The rheometer was equipped with a 20 mm diameter stainless steel cone with a truncation gap of 26 μm and 1° cone angle. The bottom plate was constituted by a Peltier element used to control the temperature with an accuracy of ± 0.1  °C.

A typical volume of 0.036 mL of sample was pipetted onto the bottom plate at 20 °C. Once the top cone was lowered to the measuring position, the sample was surrounded by a low-viscosity mineral oil to reduce water evaporation. Rapid experiments with no oil at room temperature corroborated the results obtained with oil. Experiments conformed to previous work [47].

The linear viscoelastic limits were probed through dynamic strain sweep experiments at 10 rad/s and 10 °C. A shear strain of 0.01 strain units confirmed the linear viscoelastic regime for the whole temperature window used in the study. The oscillatory temperature ramp experiments were performed from 10 to 40 °C with a frequency of 10 rad/s and a strain of 0.01 confirmed by the dynamic strain sweep test. The heating rate was 0.5 °C/min. The dynamic time sweep experiments were performed from 25 to 37 °C at 10 rad/s with the strain of 0.01 to determine the time evolution of viscoelastic response of solutions. After the gel was formed and stabilized, another dynamic time sweep experiment was performed from 37 °C to 25 °C at the same frequency and strain to test the reversibility of gelation. The monitored rheological functions were: storage modulus G′ (elastic contribution to the material response), loss modulus G″ (the viscous contribution to the material response), and ratio G″/G′, or the loss factor tan(δ).

### Mouse model of sciatic nerve crush Injury

Sciatic nerve (SN) crush injury was performed as previously described with pressure-gauge-tethered forceps [18, 20]. Briefly, after intraperitoneal (IP) ketamine (100 mg/kg)/xylazine (10 mg/kg) anesthesia, the right hindlimb was shaved and prepped with alcohol and povidone-iodine (Betadine). Under a binocular microscope (Model PZMIII, World Precision Instruments), a lateral skin incision (~ 2.5 cm) was made along the length of the femur and the sciatic nerve (SN) was exposed through the iliotibial band. Crush injury was performed ~ 3 mm proximal to the SN trifurcation using calibrated forceps (3 mm tip width, 18–1107, Miltex Instruments, York, PA) for 30 s duration at a pressure of 4.4 MPa. The incision was closed with surgical staples and mice were given post-operative slow release buprenorphine (0.05 mg/kg, subcutaneous) as an analgesic. The experimental animals (n = 5/group) were randomly assigned to Sham (normal saline, 0.1 ml/mouse, intraperitoneal (IP)), SN crush injury with saline (normal saline, 0.1 ml/mouse, IP), SN crush injury with PLGA-PEG vehicle (~ 50 µL on sciatic nerve injury site), and SN crush injury with EPO-PLGA-PEG (0.5 IU/µL EPO concentration, ~ 50 µL on sciatic nerve injury site) groups. Systemic EPO was given IP immediately after injury and on days 1 and 2 post-injury. Local administration groups received thermogel immediately after crush injury. The animals were euthanized on post-injury day 21 to harvest sciatic nerves for histological analysis.

### In vivo degradation of EPO-PLGA-PEG and EPO release in mice

Blood was retro-orbitally collected at various timepoints (days 0, 3, 7, 14, and 21) to sample serum via centrifugation (15 min, 1500 rpm, 4 °C) after leaving the samples on ice for 30 min. Serum EPO concentration was determined using ELISA, as previously described (see section “Quantification of EPO Release from Hydrogels”). To assess in vivo biodegradation of EPO-PLGA-PEG, the sciatic nerves were surgically exposed at weekly timepoints to observe location, adherence to the nerve, and mass of the gel.

### Hematological evaluation

Mice were anesthetized using isoflurane (IsoSol™, VEDCO). Retroorbital blood samples (~ 100 µL) were collected into K_2_EDTA anticoagulant tubes (07 601, Safe-T-Fill, RAM Scientific) using heparinized microhematocrit capillary tubes (22-260950, Fisher Scientific). Blood samples were immediately processed for hematological evaluation to determine hemoglobin level (Hb, g/dl) using an automatic blood cell counter (Element HT5 Veterinary hematology analyzer).

### Sciatic function index (SFI)

To study global motor functional recovery, SFI was determined by walking track analysis as previously described [18, 20]. Briefly, mice were trained to walk freely along a 77 cm by 7 cm corridor lined with paper and individual footprints of the hindlimbs were obtained before surgery as baseline and on post-surgery days 3, 7, 14, and 21. Two blinded observers measured three footprints per hindlimb with digital calipers. SFI was calculated using three parameters: (1) toe spread (TS, first to the fifth toe), (2) total print length (PL), and (3) intermediate toe spread (IT, second to the fourth toe) and the following formula: SFI = − 38.3 {(EPL-NPL)/NPL} + 109.5 {(ETS-NTS)/NTS} + 13.3{(EIT-NIT)/NIT} − 8.8, where E is for experimental (injured) and N is for normal (contralateral uninjured) sides.

### Hindlimb grip strength test

To quantify muscular strength, a grip strength meter (BIO-GS3, Bioseb-In Vivo Research Instruments, Pinellas Park, FL) was used to measure hindlimb grip force [20, 48]. The mice were restrained by holding the scruff and base of the tail. Mice were allowed to hold the grid and were gently pulled along the length of the sensor grid until the grip was released. The maximal peak force value was recorded 5 times per animal to calculate the average grip strength. Attention was paid to minimize paw injury and habit formation during each trial.

### Von Frey Test

To assess sensory recovery after injury, mice were placed in a transparent polycarbonate chamber (~ 10 × 10 cm) with a metallic mesh floor approximately 25 cm above a table. Animals were acclimatized for approximately 30 min prior to testing. Sensory nerve testing was performed as previously described using von Frey filament unit (NC12775-08, Touch Test® Sensory Evaluators) [48, 49]. The 1 g force filament was applied to the plantar surface of the hindlimb through the mesh floor. The animal withdrawing its paw was considered a positive response and the withdrawal reflex of the hindlimb was recorded five times per animal to calculate the average percent response.

### Whole-Mount immunostaining of sciatic nerves

After SFI analysis on post-surgery day 21, whole sciatic nerves were collected and fixed for 5 h in 4% paraformaldehyde at 4 °C. Macroscopically, the nerves were found in good continuity. Nerves were washed in PBS with 1% Triton X-100 (PTX) and incubated in blocking solution (10% normal goat serum, Jackson Immunoresearch, in 5% BSA PTX) overnight at 4 °C. On the following day, nerves were transferred into primary antibodies in 5% BSA PTX and incubated for 72 h at 4 °C with gentle rocking. Primary antibodies were NF-H (1:1000; NB300-135, Novus Biologicals), MPZ (1:500; PZ0, Aves Labs), and CD31 (1:100; 553,370, BD Pharmingen). Nerves were then washed with PTX every hour for 4 h at 4 °C. After PTX washes, nerves were incubated with Alexa Fluor 488 (1:500, A11008, Invitrogen), 594 (1:500, A11042, Invitrogen), and 647 (1:500, A21247, Invitrogen) secondary antibodies for 48 h at 4 °C with gentle rocking. Next, nerves were washed in PTX three times, followed by 1-h PTX washes for 4 h. Nerves were then washed overnight in PTX at 4 °C. Next day, nerves were washed with PBS for the removal of triton and cleared sequentially in 25% and 50% glycerol (G6279, Sigma) in PBS for 6 and 12 h, respectively. Following clearing, nerves were mounted in SlowFade Gold Antifade Mountant with DAPI (S36939, Invitrogen). Stained whole nerves were imaged using ZEISS Axio Observer 7 equipped with an Apotome.2 (Carl Zeiss Microscopy GmbH, Jena, Germany). Tiling and z-stack functions were used to image whole nerve. Maximum intensity projection was used to pull the data from all Z-stacks and represent it as a 2-D image [50, 51].

### Quantitative analysis of nerve fibers and blood vessels

Nerve images were captured at different depths using Z-stacking and this 3-D data was pulled together as a 2-D image using maximum intensity projection. The use of this maximum intensity projected image ensures counting the same fiber at different depths once. For quantitative analysis of whole-mount images, each nerve was divided into three zones: proximal (defined as all regions immediately proximal to the 3 mm injury site), injury site (3 mm crush injury), and distal (all regions immediately distal to the injury site and including the trifurcation). For each nerve in each zone, ImageJ was used to quantify integrated density in each region. For analysis of CD31-stained images, we studied angiogenesis using AngioTool (version 0.6a, National Cancer Institute) [52]. We analyzed vessel density and branching using this software which provides automated measures of vessels.

### Quantification of percent myelinated fibers

Sciatic nerve processing and immunohistochemical staining were performed as previously described with slight modification [18, 20]. SNs were harvested on day 21 post-injury from the ipsilateral hindlimbs of mice. Nerves were fixed in 4% paraformaldehyde (PFA) solutions overnight, washed with 70% alcohol, and embedded in paraffin. A microtome (Model RM2235, Leica, Buffalo Grove, IL) was used to cut serial 5 μm transverse sections from the paraffin blocks. Tissue sections were deparaffinized, serially rehydrated with xylene and ethanol, and antigen retrieval was performed using 10 mM sodium citrate buffer (pH 6.0). Permeabilization and nonspecific binding blocking were done using 1% Triton X-100 and 5% goat serum, respectively. Primary antibody staining was performed with anti-NF-H (1:1000; NB300-135, Novus biologicals) and anti-MPZ (1:1000; PZ0, Aves Labs) followed by secondary antibody incubation with Alexa Fluor 488 (1:1000; A11008, Invitrogen) and Alexa Fluor 647 (1:1000; A21449, Invitrogen). Staining without primary antibodies served as a control for non-specific fluorescence. Nuclei were counter-stained with ProLong™ Gold antifade reagent with DAPI (P36935, Invitrogen) and sections were observed under a fluorescent microscope (ZEISS Apotome 2). Percent of myelinated fibers was calculated by counting the number of myelinated axons divided by the total number of axons.

### Statistical analysis

All data were analyzed using GraphPad Prism Version 8.4.3 (San Diego, USA). All the results were expressed as mean ± standard error of the mean (SEM). For group comparison, the statistical differences of mean values were analyzed by unpaired t-tests, one-way, and two-way analysis of variance (ANOVA). A p-value of less than 0.05 was considered as significant.

## Supplementary Information

**Additional file 1: Figure S1**. EPO ELISA standard curve used for EPO concentration calculation. Linear regression was used to yield a line of best fit with an R2 value of 0.9897.** Figure S2**. AngioTool reconstruction images clearly depict the blood vessel architecture as red lines and their branching points as blue dots.

## Data Availability

Not applicable.

## Abbreviations

TPNI: Traumatic peripheral nerve injury
EPO: Erythropoietin
PEG: Polyethylene glycol
PLGA: Poly lactic acid-co-glycolic acid
IP: Intraperitoneal
SN: Sciatic nerve
SFI: Sciatic function index
WTA: Walking track analysis
NF-H: Neurofilament-H
MPZ: Myelin protein zero
