# Supplementary material for: Erythropoietin-PLGA-PEG as a local treatment to promote functional recovery and neurovascular regeneration after peripheral nerve injury
Source: J Nanobiotechnology. 2022 Oct 28;20:461. doi: 10.1186/s12951-022-01666-5 (PMC9617443; doi:10.1186/s12951-022-01666-5)
Supplement: Supplementary file 1 — Additional file 1: Figure S1. EPO ELISA standard curve used for EPO concentration calculation. Linear regression was used to yield a line of best fit with an R2 value of 0.9897. Figure S2. AngioTool reconstruction images clearly depict the blood vessel architecture as red lines and their branching points as blue dots. [file 12951_2022_1666_MOESM1_ESM.docx]

**Supplemental Figures**

**
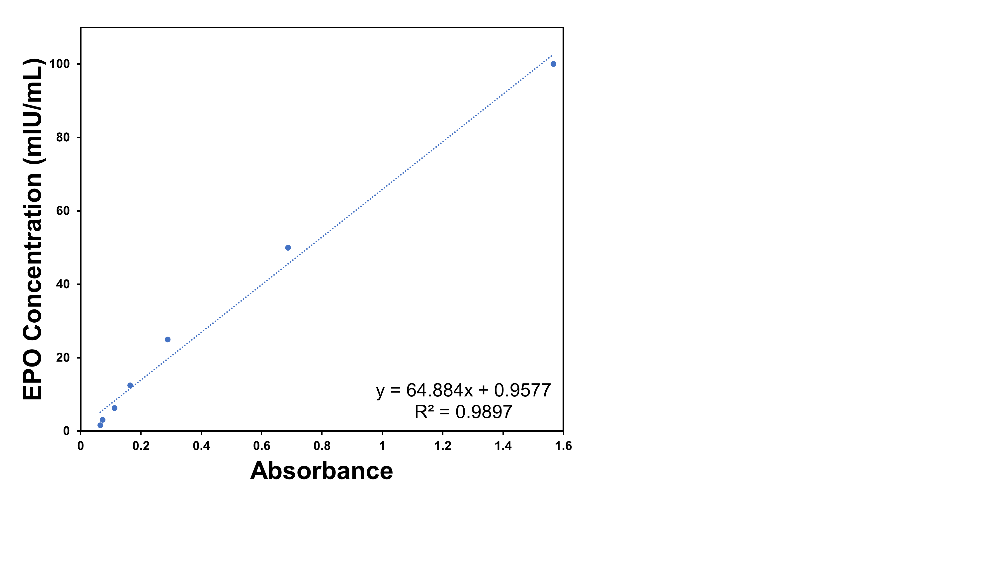
**

**Figure S1.** EPO ELISA standard curve used for EPO concentration calculation. Linear regression was used to yield a line of best fit with an R^2^ value of 0.9897.

**
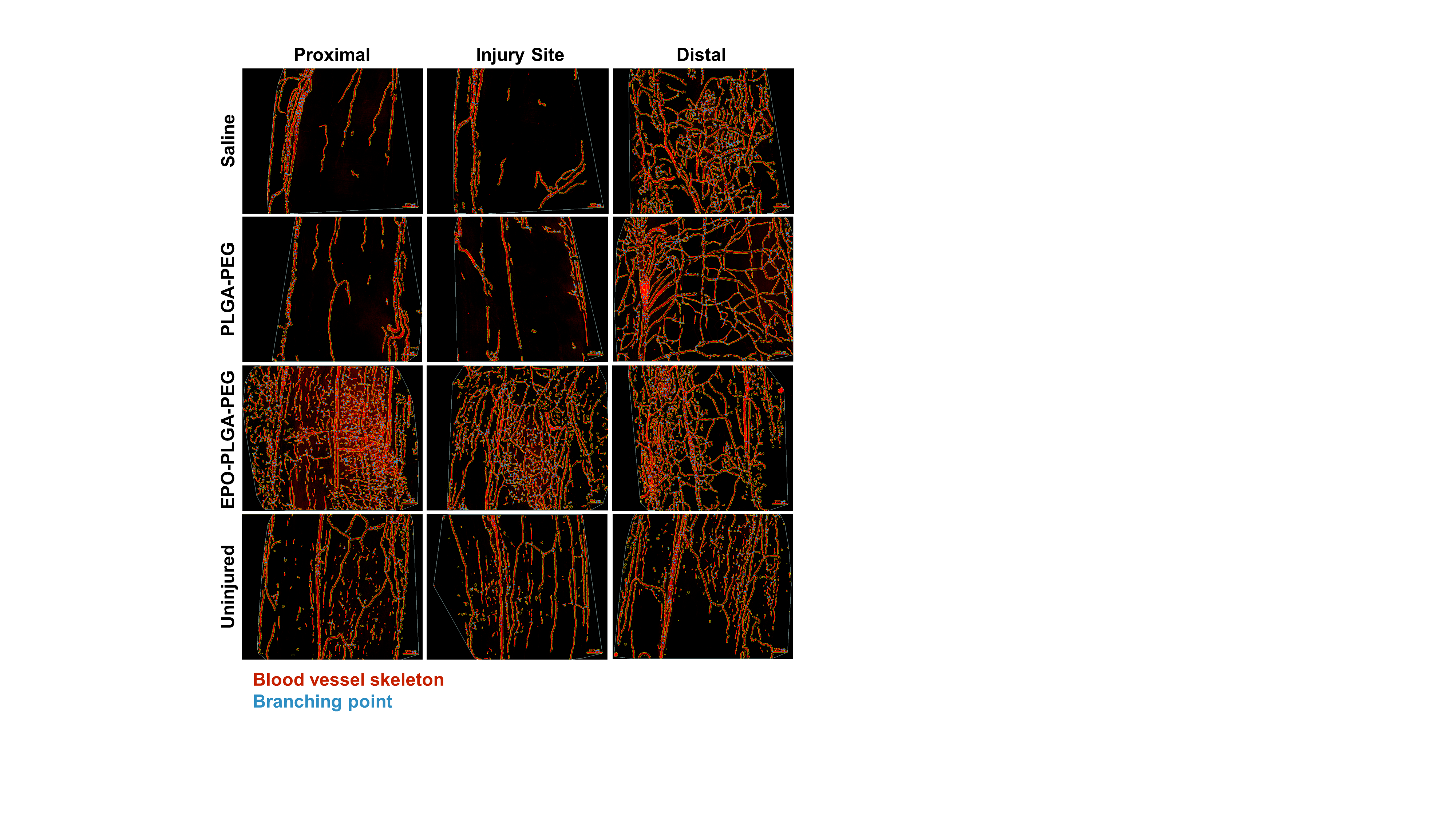
**

**Figure S2.** AngioTool reconstruction images clearly depict the blood vessel architecture as red lines and their branching points as blue dots.
